# Immune expression signatures as candidate prognostic biomarkers of age and gender survival differences in cutaneous melanoma

**DOI:** 10.1038/s41598-020-69082-z

**Published:** 2020-07-23

**Authors:** Yi-Jun Kim, Kyubo Kim, Kye Hwa Lee, Jiyoung Kim, Wonguen Jung

**Affiliations:** 1 Department of Radiation Oncology, Ewha Womans University College of Medicine, 1071 Anyangcheon-ro, Yangcheon-gu, Seoul, 07985 Republic of Korea; 2 Institute of Convergence Medicine, Ewha Womans University Mokdong Hospital, Seoul, 07985 Republic of Korea; 3 Biomedical Informatics Department, Asan Medical Center, Seoul, 05505 Republic of Korea

**Keywords:** Cancer, Computational biology and bioinformatics, Genetics, Immunology, Diseases, Oncology

## Abstract

This study aims to investigate the difference of gene expression and its prognostic significance in younger women with melanoma. Significantly upregulated genes in tumors compared to normal skin tissues were extracted. Among these genes, genes that significantly affected survival according to expression level were selected, and pathway annotation was performed. The patient proportion with high/low expression of the most significant pathways was analyzed in each age (< 50, 50–59, ≥ 60) and gender group. Survival was analyzed according to age, gender, and pathways. The most significant pathways that were upregulated in tumor tissues and also had impacts on survival were programmed cell death protein [PD]-1, interferon-γ, and interferon-α/β pathways. In women, the immune signaling rate in patients was higher than men and decreased with age (63.5%, 53.8%, and 47.6%). In men, the decreasing tendency was minimal (47.6%, 50.0%, and 41.6%). In patients aged < 60 years, women had a favorable survival rate than men (*p* = 0.055). Except for patients with high immune signaling, no survival difference was observed between genders (*p* = 0.6). In conclusion, younger female melanoma patients had high immune signaling than older women and men. This immune signaling improved survival of the younger female patients.

## Introduction

In 2018, the global incidence of melanoma of the skin was 1.6% of all cancers, a similar rate with cancer in the brain/nervous system or ovarian cancer^1^. In the United States, the estimated numbers of new cases of melanoma in men and women in 2019 were 57,220 and 39,260, respectively, and the estimated number of new deaths in men and women was 4,740 and 2,490, respectively^2^.

Female melanoma patients have favorable primary tumor characteristics^3^, lower risk of metastasis^4^, and longer survival^3^ than male patients. Moreover, a more favorable prognosis in younger patients is more pronounced in women^5^.

The reason for gender and age differences in the survival rate in melanoma remains unclear. Innate gender differences such as sex hormones, immune system, oxidative stress, and environmental differences, such as tanning bed exposure have been identified as possible reasons for the sexual disparity in melanoma prognosis^6^.

Until now, genetic analyses for the survival disparity between genders in melanoma have been performed^7^. Several studies reported that the differences in pigmentation^8^, apoptosis^9^, reactive oxygen species^10^, or sex-specific pleiotropic cancer-related genes are likely to contribute to the prognostic differences between genders. Another study focused on the DNA mutational burden difference between genders in metastatic melanoma^7^. However, these studies did not analyze the patients’ age, which is especially related to the menopausal status of women. To evaluate the causes of the prognostic difference according to age as well as gender, transcriptome analysis rather than mutational or single nucleotide polymorphisms (SNPs) analysis may be more reasonable.

In this study, we confirmed a favorable prognosis in younger female patients of melanoma compared to older women and young men with melanoma by using the Surveillance, Epidemiology, and End Results (SEER, RRID: SCR_006902) registry. After that, we analyzed the differences in mRNA expression between tumor and normal tissues and identified genetic pathways that affect survival. We investigated the infiltration degrees and survival impact of these pathways according to age and gender. The results of this study will provide a deeper understanding of melanoma and insight into new therapeutic approaches.

## Results

### SEER database analysis

**Figure 1:**
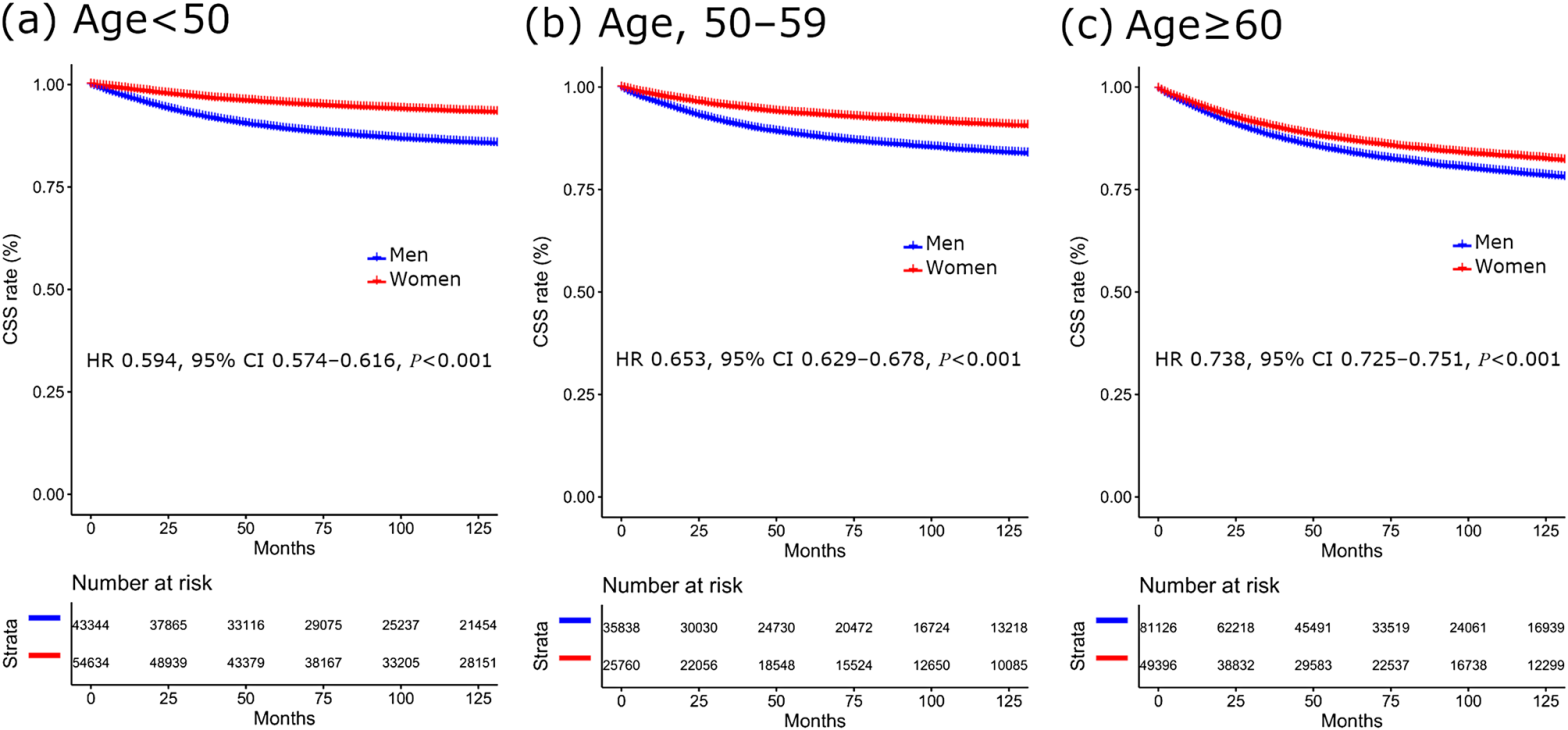
Comparison of CSS in melanoma according to age and gender. (**a**) CSS in patients aged < 50, (**b**) CSS in patients aged 50–59, and (**c**) CSS in patients aged ≥ 60. CSS, cancer-specific survival; HR, hazard ratio; CI, confidence interval.

In the SEER database analysis, the total number of patients was 290,098. In a comparison of the impact of gender on cancer-specific survival (CSS) rate in each age group, CSS rates were better in women than men regardless of the age groups. The 5-year CSS rates for men and women were 89.4% versus 95.5%, 88.1% versus 93.4%, and 84.1% versus 87.1% in the aged < 50, 50–59, and ≥ 60 groups, respectively (*p* < 0.001, all). In multivariate analyses, female melanoma patients had significantly higher CSS rates than men in all age groups (in the aged < 50 group, hazard ratio [HR], 0.594, 95% confidence interval (CI) 0.574–0.616, *p* < 0.001; in the aged 50–59 group, HR, 0.653, 95% CI 0.629–0.678, *p* < 0.001; and in the aged ≥ 60 group, HR, 0.738, 95% CI 0.725–0.751, *p* < 0.001). Better prognosis for women was more prominent in the younger groups compared to the older groups with a lower HR (HR, 0.594, 95% CI 0.574–0.616 vs. HR, 0.653, 95% CI 0.629–0.678 vs. HR, 0.738, 95% CI 0.725–0.751) (Fig. 1).

### Genes that significantly impact survival according to the expression level

The mRNA expression levels of melanoma tumor tissues in The Cancer Genome Atlas Program (TCGA, RRID: SCR_003193) database and normal skin tissues in the Genotype-Tissue Expression (GTEx, RRID: SCR_013042) database were compared. The numbers of melanoma patients in the TCGA and database were 470 and 974, respectively.

In the analysis for all patients, a total of 1,021 genes were significantly upregulated in the tumor tissues compared to normal skin tissues with log2-fold change (logFC) > 1.5 and false discovery rate (FDR) Q < 0.01. Among these genes, 209 genes significantly affected survival with *p* < 0.001. Heatmaps of gene expression of the 1,021 and 209 genes showed that the 209 gene expressions were predominant for young female patients (Supplementary Fig. S1a, b).

In the functional analysis, the top-ranked functions of these 209 genes were immune-related functions such as interferon (IFN) and programmed cell death protein (PD)-1 signaling (PD-1 signaling [R-HSA-389948], FDR = 2.38e−14; IFN-γ signaling [R-HSA-877300], FDR = 2.38e−14; IFN-α/β signaling [R-HAS-909733], FDR = 1.53e−04).

**Figure 2:**
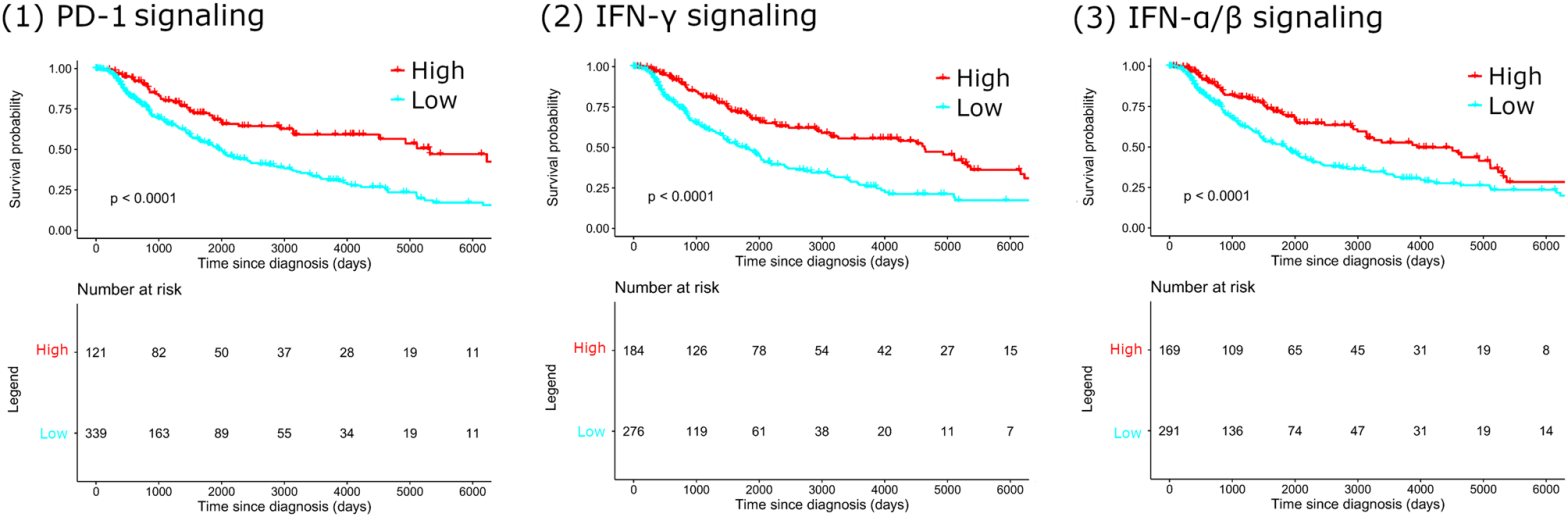
Comparison of OS in melanoma according to signaling expression level. (**1**) PD-1 signaling, (**2**) IFN-γ signaling, and (**3**) IFN-α/β signaling. PD-1, programmed cell death protein 1; IFN, interferon; OS, overall survival.

Upregulations of the PD-1, IFN-γ, IFN-α/β pathways were correlated with favorable survival in melanoma (Table 1). Patients with upregulated signaling were defined as patients with at least one or more genes with high expression (above the mean of the top third) of specific signaling. Patients with downregulated signaling were defined as patients with no genes with high expression of specific signaling. The median survival with high/low PD-1, IFN-γ, and IFN-α/β signaling expressions were 175 versus 64 months, 152 versus 59 months, and 103 versus 48 months, respectively (*p* < 0.001, all) (Fig. 2).

**Table 1:** Survival comparison according to the expression level of immune pathway-related genes that are upregulated in melanoma tissues than normal skin tissues and affect survival according to the expression level.

Pathway	Genes	Top third expression (> 67%)			Bottom third expression (< 33%)			*p*-value^a^
		Observed deaths (no.)	Expected deaths (no.)	Total patients (no.)	Observed deaths (no.)	Expected deaths (no.)	Total patients (no.)	
PD-1 signaling (R-HSA-389948)	HLA-DPB1	53	82.3	153	89	59.7	156	0.000001
	HLA-DRB1	55	84.0	153	86	57.0	154	0.000001
	HLA-DRA	54	81.9	153	83	55.1	155	0.000001
	HLA-DPA1	56	83.5	153	85	57.5	156	0.000002
	HLA-DRB5	50	75.9	153	89	63.1	154	0.000008
	HLA-DQB1	51	76.7	153	88	62.3	154	0.000010
	CD3E	58	82.8	151	88	63.2	155	0.000030
	HLA-DQA1	59	82.7	153	81	57.3	151	0.000042
	CD4	66	89.0	153	78	55.0	154	0.000065
	HLA-DQA2	60	81.4	150	86	64.6	154	0.000329
IFN-γ signaling (R-HSA-877300)	GBP4	57	92.8	154	86	50.2	153	< 0.000001
	GBP1	57	88.4	154	84	52.6	152	< 0.000001
	GBP5	60	91	153	89	58.0	154	< 0.000001
	HLA-DPB1	53	82.3	153	89	59.7	156	0.000001
	HLA-DRB1	55	84.0	153	86	57.0	154	0.000001
	IRF1	55	84.0	153	88	59.0	154	0.000001
	HLA-DRA	54	81.9	153	83	55.1	155	0.000001
	HLA-DPA1	56	83.5	153	85	57.5	156	0.000002
	HLA-DRB5	50	75.9	153	89	63.1	154	0.000008
	HLA-DQB1	51	76.7	153	88	62.3	154	0.000010
	ICAM1	61	85.9	152	82	57.1	153	0.000018
	HLA-DQA1	59	82.7	153	81	57.3	151	0.000042
	OAS1	62	85.7	153	83	59.3	153	0.000056
	OAS2	60	82.7	153	83	60.3	151	0.000118
	IRF8	66	89.0	152	85	62.0	155	0.000128
	VCAM1	74	96.3	153	80	57.7	153	0.000175
	HLA-DQA2	60	81.4	150	86	64.6	154	0.000329
	IFI30	62	82.6	150	80	59.4	155	0.000419
IFN-α/β signaling (R-HSA-909733)	BST2	50	79.4	150	85	55.6	154	< 0.000001
	IRF1	55	84.0	153	88	59.0	154	0.000001
	PSMB8	57	82.1	152	88	62.9	155	0.000022
	IFIT3	67	91.9	153	83	58.1	151	0.000025
	IFI27	56	80.8	151	91	66.2	153	0.000033
	OAS1	62	85.7	153	83	59.3	153	0.000056
	OAS2	60	82.7	153	83	60.3	151	0.000118
	IRF8	66	89.0	152	85	62.0	155	0.000128
	IFI35	57	78.8	153	86	64.2	153	0.000205
	IFIT1	68	88.3	153	73	52.7	150	0.000353

^a^Log-rank test.

Genes that were downregulated in tumors than normal skin tissues (logFC < − 1.5 and FDR Q < 0.01) and affected survival (*p* < 0.001) were also analyzed using the same methods. A total of 2,381 genes were significantly downregulated. Among them, 123 genes significantly affected survival. Heatmaps of gene expressions for the downregulated genes showed that gene expressions were not significantly different among the age and sex groups (Supplementary Fig. S1c, d). In functional analysis, the PD-1, IFN-γ, and IFN-α/β signaling were not observed.

### Immune signaling according to age and gender

**Figure 3:**
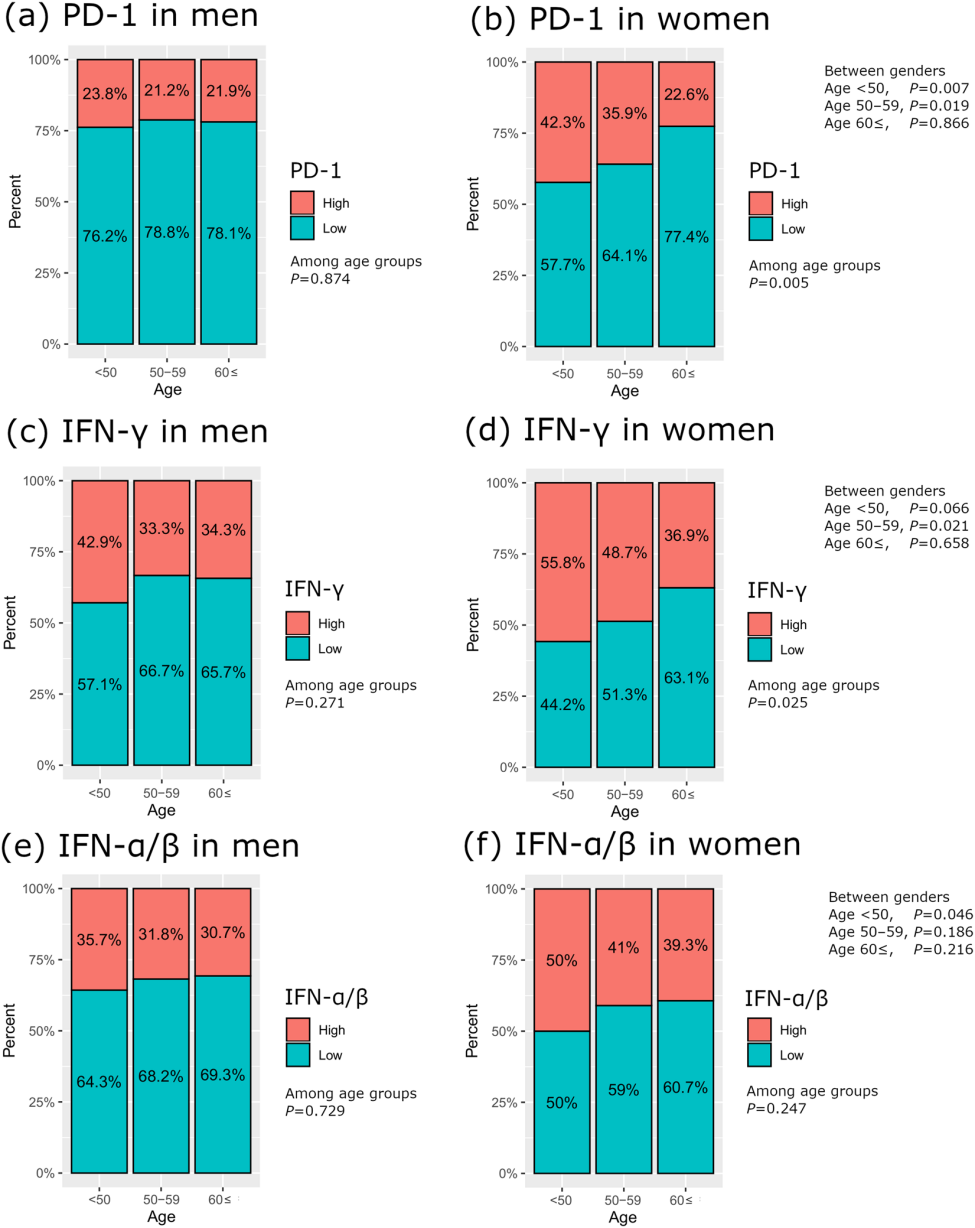
Immune infiltration according to age and gender in melanoma. PD-1, programmed cell death protein 1; IFN, interferon. *p* value, Pearson's chi-squared test.

The expression level of each immune-related signaling was evaluated according to age and gender (Fig. 3). The high expression group was defined as a group of patients with one or more genes with high expression of specific immune-related signaling. The low expression group was defined as a group of patients with no genes with high expression of specific immune-related signaling.

In women, the proportion of patients with high expression levels of PD-1, IFN-γ, and IFN-α/β signaling gradually decreased with age (Chi-squared test, *p* = 0.005, 0.025, and 0.247, respectively), while this trend was not evident in men (Chi-squared test, *p* = 0.874, 0.271, and 0.729, respectively). The proportion of patients with high immune signaling was greater in women than men in the younger age groups, and in the aged 60 ≤ group, the difference in immune signaling between genders was minimal (Fig. 3). The proportion of patients with high immune signaling (PD-1, IFN-γ, and IFN-α/β, all) in the aged < 50, 50–59, and ≥ 60 groups were 63.5%, 53.8%, 47.6% in women, and 47.6%, 50.5%, 41.6% in men.

### Survival comparison according to age, gender, and immune signaling

**Figure 4:**
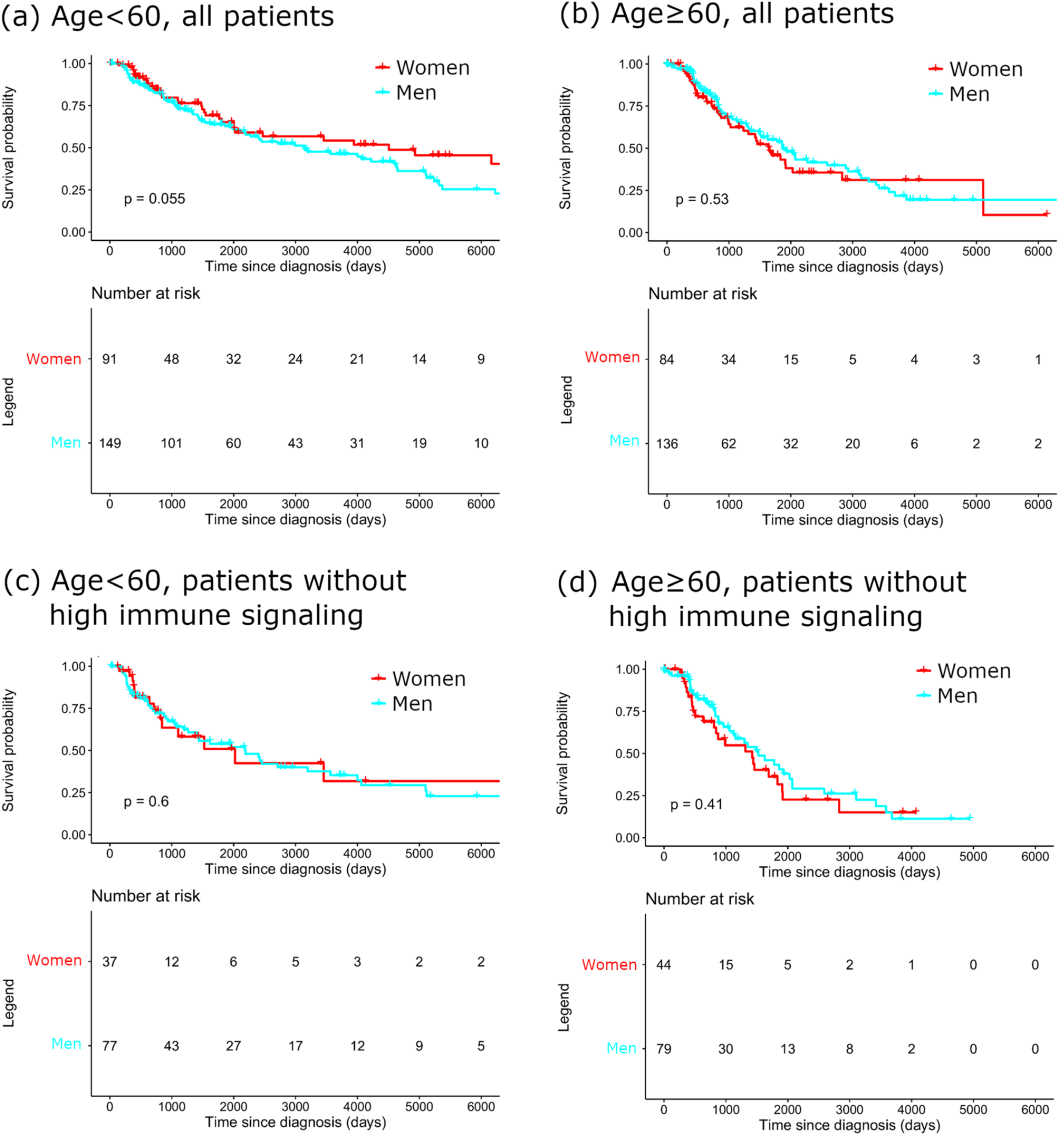
Comparison of OS in melanoma according to age, gender, and immune infiltration. (**a**) OS in patients aged < 60 years, (**b**) OS in patients aged ≥ 60 years, and (**c**) OS in patients aged < 60 without high immune signaling. (**d**) OS in patients aged ≥ 60 without high immune signaling. OS, overall survival.

The effect of immune signaling levels on survival was assessed by gender and age (Fig. 3). Patients with high immune signaling were defined as patients with at least one or more genes with high expression of PD-1, IFN-γ, or IFN-α/β signaling. Patients with low immune signaling were defined as patients with no genes with high expression of PD-1, IFN-γ, or IFN-α/β signaling. In the patients < 60 years of age, women had a favorable overall survival (OS) trend than men (median survival in men and women, 103 vs. 148 months, *p* = 0.055). In the patients aged 60 ≤ years, women did not show better OS than men (median survival in men and women, 72 vs. 66 months, *p* = 0.53). When excluding patients with high levels of immune signaling, survival differences by gender in the < 60 years old group disappeared (median survival in men and women, 62 vs. 54 months, *p* = 0.6). It suggests that the high level of immune signaling observed in women under 60 years old may be one of the reasons for the excellent survival of female melanoma patients (Fig. 4).

We analyzed the impact of treatment on survival. Of the 470 patients in the TCGA database, 25 received treatment prior to biopsy, and 251 patients underwent treatment after biopsy (radiotherapy or chemotherapy). Twenty of the 25 patients who were treated before biopsy were also treated after biopsy. We evaluated the impact of treatment on survival in all patients, patients aged < 60 years and patients aged ≥ 60 years, respectively. In the analysis of each group, treated patients had a favorable prognosis for survival. However, none of the treatment variables significantly affected survival (Supplementary Table S1–S3). In addition, the distribution of these treatment variables between gender groups was not significantly different (Supplementary Table S4–S6), suggesting that the treatment variables in the TCGA data may not affect survival differences between male and female melanoma patients.

### Validation

To confirm the impact of immune signaling on survival according to age and gender, another melanoma dataset (European Molecular Biology Laboratory-European Bioinformatics Institute [EMBL-EBI], accession number: E-GEOD-65904) was analyzed (Supplementary Fig. S2). The number of melanoma patients was 214. Immune-related signaling was the most pronounced function of the genes which significantly related to survival. Although not always statistically significant, possibly because of relatively small sample size, immune signaling was more prominent in women than in men. The immune signaling difference between genders decreased with age (Supplementary Fig. S3). When excluding patients with high immune signaling, women's favorable survival tendency was reduced (Supplementary Fig. S4).

To improve the homogeneity of the patient group, only patients with metastatic melanoma in the TCGA database were reanalyzed. The results were similar to those of all patients with primary or metastatic melanoma (Supplementary Figs. S5, S6, and S7).

The gene expression of normal skin tissue can be different from melanocyte. Instead of normal skin tissue (GTEx database), gene expressions of melanocytes (n = 11) from the Gene Expression Omnibus database (GEO, RRID: SCR_005012) were compared with those of melanoma tissues. The results were similar to the comparison between normal skin and melanoma tissues (Supplementary Figs. S8, S9, and S10).

## Discussion

### In summary

In melanoma, women compared to men, and younger women compared to older women had significantly favorable survival rates. Among genes that were upregulated in tumor tissues (TCGA database) compared to normal skin tissues (GTEx database), genes that were related to immune signaling (PD-1, IFN-gamma, and IFN-*α/β*) most significantly affected survival. The proportion of patients with upregulated immune signaling in female patients was higher than male patients. However, the proportion decreased with age in women, resulting in minor differences between genders in patients aged ≥ 60 years. In patients aged < 60 years, female patients had a favorable survival rate than male patients. When patients with high immune signaling were excluded, the survival superiority in women was not observed. Therefore, the favorable prognosis of women aged < 60 years may be associated with upregulated immune-related processes compared to women aged ≥ 60 years and men in melanoma.

### Gender, age, and immune signaling in melanom*a*

In this study, we identified the gene functions that are upregulated in tumors than normal tissues and also influence the survival rate in melanoma. The first assumption was that upregulated signaling in tumors than normal tissue which has an impact on survival would be associated with poor prognosis related to recurrence or metastasis of the disease. In the overall gene expression, it has been known that the gene signaling most affecting the survival rate in melanoma is immune-related signaling^11^. In our study, we found that this tendency was the same in analyzes using only genes that were upregulated in tumors than normal tissues. It suggests that the stronger the immune response induced by cancer, the higher the survival rate of melanoma.

The median menopausal age of women is approximately 50–52 years, with 95% of women experiencing the last menstrual period between 44–56 years^12^. In our analysis using a big cohort dataset, women had a higher survival rate than men, with the difference gradually decreasing with age. Since much of the difference between women and men mainly derived from differences in sex hormones, the difference in survival rate is also likely to be the effect of sex hormones^13,14^. However, the effects of sex hormones on melanoma are complex. One study showed that estradiol promoted the production of IFN-α/β, suggesting that estrogen may contribute to the gender differences in immune pathways^15^. Another study demonstrated that estrogen therapy increased the risk of melanoma, while progestin therapy reduced the risk^14^. Another experimental study reported that estrogen and progesterone have no protective function against cancer, but testosterone replacement therapy reduced the burden of metastatic lung tumors in mice melanoma^13^. Therefore, sex hormone alone may not be enough to explain the difference in immune signaling between pre- and post-menopausal women and between men and women. Our study showed the effect of immune signaling levels on survival by age and gender. Therefore, a more direct immunological approach may be more effective in improving the prognosis for older female and male melanoma patients than the sex hormone-based approach.

### PD-1, IFN-γ, and IFN-α/β signaling in melanoma

In our study, the expression levels of the PD-1, IFN-γ, and IFN-α/β pathways correlated significantly with patients’ prognosis. Several studies demonstrated that the upregulation of PD-1, IFN-γ, and IFN-α/β pathways are associated with higher immune infiltration rates. Expression of each of PD-1 and PD-L1 was significantly correlated with higher tumor-infiltrating lymphocytes (TIL) expression and pathological complete response^16,17^. Nirschl et al.^18^ studied melanoma cells and found that IFN-γ induces a steady-state program in which immune mononuclear phagocytes share during differentiation and entry into healthy tissue. IFN-α/β activates natural killer (NK) cells, antigen-presenting dendritic cells (DC), CD4, and CD8 T cells. For the acquisition of effector functions in T cell, signaling through the IFN-alpha receptor (IFNAR) is critical. IFN-α/β also increases antigen presentation of tumor cells to be recognized by T cells^19,20^.

Of these, PD-1 and IFN-α/β pathways are already being used as therapeutic targets for melanoma. Nivolumab and Pembrolizumab are anti-PD-1 agents and the primary treatment for advanced, metastatic, or unresectable melanoma^21–23^. Adjuvant IFN-α has been widely used for decades in melanoma^24^. One study showed that IFN-α and IFN-β have different antitumor effects—IFN-α2b inhibits tumor growth and lymph node metastasis, while IFN-β1a has more potent antiproliferative and proapoptotic effects^25^.

In our study, IFN-γ was one of the most pronounced functions for genes that were upregulated and they produced a significant impact on survival in melanoma. IFN-γ is a cytokine that plays a pivotal role in anti-tumor host immunity produced predominantly by NK cells and T-cells^26,27^. Several studies demonstrated that high expression of the IFN-γ response signature in tumor tissues was associated with long-term benefit from ipilimumab treatment, and melanoma tumors with loss of IFN-γ signaling lack in response to ipilimumab^28^. Also, IFN-γ receptor signaling pathways regulate PD-L1 and PD-L2 expressions^29^, and patients with enriched mutations in the IFN-γ receptor signaling pathways did not respond to anti-PD-1 therapy^30^. Therefore, upregulated IFN-γ response signaling may represent high sensitivity to immune checkpoints inhibitors as well as an active immune system of the host.

### Limitations of this study

Our study had several limitations. We combined the TCGA and GTEx databases to compare mRNA expression levels between tumor (TCGA database) and normal skin tissues (GTEx database) because of the limited number of normal skin tissues in the TCGA database. We used the *recount2* pipeline, which enables multiple downstream analysis including the GTEx and TCGA databases^30,31^. Although all *recount2* tissues have been processed with a single pipeline, batch effects among different datasets could still occur and should be considered in downstream analyses. The melanoma patients were classified according to age (< 50, 50–59, and ≥ 60 years). In the absence of menopausal information based on hormone testing results, patient/individual groups were divided based on age 50 and 60 to reflect women’s menopausal status as much as possible^12^.

Albeit these limitations, our study may provide meaningful insight regarding prognostic differences according to the immune signaling level between the age and gender groups in melanoma. These results may contribute to more precise management for melanoma patients.

## Methods

### Survival comparison between gender and age groups using the SEER database

Before mRNA analysis, the SEER database was analyzed to confirm the prognostic value of age on survival in melanoma. The SEER database is an authoritative cancer registry of the National Cancer Institute (NCI) in the USA. Patients who were diagnosed with melanoma (International Classification of Diseases for Oncology [ICD-O]-3 histology 8,720–8,780) of the skin (C440-449) from 1975 to 2015 in the database were selected. Only pathologically confirmed and primary malignancy cases were included. Patients without survival time or cause of death information were excluded from the analysis. Information on sex, age at diagnosis, race, year of diagnosis, tumor grade, histology, surgery, chemotherapy, radiotherapy, insurance, and marriage were gathered.

CSS was defined as the time from diagnosis to either death caused by melanoma or the last follow-up as estimated with the Kaplan–Meier method. At first, the CSS comparisons between genders in each age group were performed using a log-rank test. Cox multivariate analysis was performed after incorporating all variables (age, race, year of diagnosis, tumor grade, histology, tumor extent, surgery, chemotherapy, radiotherapy, insurance, and marriage information) in each age group. The hazard ratio (HR) of the gender in each age group was compared.

### mRNA expression data preparation

The mRNA expression data of skin cutaneous melanoma tissue was obtained from the TCGA (RRID: SCR_003193)-human skin cutaneous melanoma project (TCGA-SKCM, primary and metastatic cancers). Gene expression quantification data (Illumina HiSeq platform) was downloaded from the legacy database (data aligned against the genome of reference hg19, GRCh37.p13) using the Genomic Data Common (GDC) Application Programming Interface (API) method.

The number of normal skin tissues was limited in the TCGA database (n = 1). For the comparison of gene expression level between tumor and normal skin tissues, we additionally used the GTEx (RRID: SCR_013042) normal skin tissue database. The *recount2* project allowed for the TCGA and GTEx data to be combined and processed under one single pipeline to avoid any technical variability affecting downstream integrative analyses^31,32^. TCGA and GTEx data downloads were performed using ‘TCGAbiolinks’ R package^33^. This package offers a function (‘TCGAquery_recount2’) to download the GTEx data as a list including a summarizedExperiment (SE) of gene counts per sample through the *recount2* project. Gene raw read count matrices (row = gene, column = sample) of the TCGA and GTEx data were combined after converting the gene identifier of the GTEx data (Ensembl ID) to the same form of the TCGA data (‘HUGO Gene Nomenclature Committee [HGNC]_symbol | Entrez ID’) using the ‘biomaRt’ R package^34^.

For this combined gene matrix of the TCGA and GTEx database, normalization, filtering, and differential expression analysis (DEA) were sequentially performed using the ‘TCGAbiolinks’ package.

### DEA, survival analysis, and pathway annotation

The mRNA expression differences between tumor (from TCGA database) and normal skin tissues (from GTEx database) were analyzed to extract significantly upregulated genes in the tumor tissues compared to normal skin tissues with logFC > 1.5 and FDR Q < 0.01.

Among the extracted genes, genes that significantly affected survival with *p* < 0.001 according to the expression level (comparison between the groups of the high threshold of 67% vs. low threshold of 33% using a log-rank test) were extracted. For survival analysis, ‘survival^35^’ and ‘survminer^36^’ R packages w ere used. Pathway annotation was performed using the Reactome (PRID: SCR_003485).

Analysis for significantly downregulated genes (logFC < −1.5 and FDR Q < 0.01) in the tumor tissues compared to normal skin tissues was also performed with the same protocol for the upregulated genes as previously mentioned.

### Validation

A database of whole-genome expression of melanoma was used as a validation set (from EMBL-EBI: E-GEOD-65904; from GEO: GSE65904). We used ‘GEOquery’ R package to download the data^37^. To change Illumina HumanHT12v4 annotation to HGNC symbol, ‘illuminaHumanv4.db’ R package was used^38^.

We also downloaded gene expression of melanocyte from the GEO database (PRID: SCR_005012) by using the ‘recount’ R package^31^. We searched all projects including ‘melanocyte’ word in their abstracts. Finally, a total of 11 samples (SRR849502, SRR1182316, SRR1182317, SRR1182319, SRR1182320, SRR1993908, SRR1993909, SRR2014614, SRR2014615, SRR2014616, and SRR2014617) from 4 projects (SRP022259, SRP039354, SRP057616, and SRP058120) were available to analyze. Gene raw read count matrices of the TCGA and the melanocyte samples were combined, and the same analysis with the TCGA and GTEx database (normalization, filtering, and DEA) was performed.

### Statistical and computational methods

R software (version 3.6.1, https://cran.r-project.org/bin/windows/base/old/3.6.1/) and its integrated development environment, RStudio (version 1.2.1335, RRID: SCR_000432) were used for all analyses.

## Conclusions

In melanoma, younger women had a significantly higher survival rate than older women and men. The younger female patients showed upregulated immune-related signaling (PD-1, IFN-γ, and IFN-α/β) compared to the older female patients and male patients, and this upregulation improved survival in melanoma. Immune signaling may serve as a prognostic marker in melanoma.

### Ethical statement

This study was exempt from ethical approval because the analysis involved only de-identified data (SEER, TCGA, and GTEx database). This study was performed in accordance with the Declaration of Helsinki.

## Supplementary information

Supplementary Information.
