# Supplementary material for: Immune expression signatures as candidate prognostic biomarkers of age and gender survival differences in cutaneous melanoma
Source: Sci Rep. 2020 Jul 23;10:12322. doi: 10.1038/s41598-020-69082-z (PMC7378165; doi:10.1038/s41598-020-69082-z)
Supplement: Supplementary file 1 — Supplementary Information. [file 41598_2020_69082_MOESM1_ESM.pdf]

Supplementary materials

Immune Expression Signatures as Candidate Prognostic Biomarkers of Age and Gender Survival Differences in Cutaneous Melanoma

Yi-Jun Kim, Kyubo Kim, Kye Hwa Lee, Jiyoung Kim, and Wonguen Jung

Supplementary Figure S1. Heatmaps of gene expression (z-score) according to age and gender in melanoma of the TCGA database. Genes in the heatmaps are listed in order of absolute log2-fold change (logFC) values.

(a-1) Heatmap of gene expressions (z-score) that were significantly upregulated in the melanoma tissue compared to normal skin tissues (n=1021)

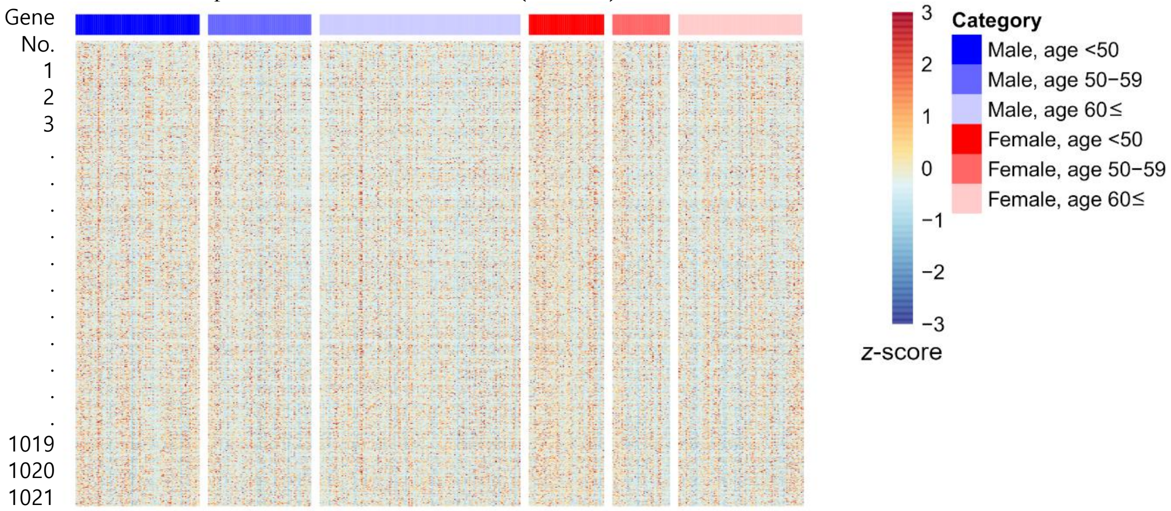

(a-2) Top 100 genes of absolute logFC among the 1021 genes

No.	Gene	logFC	FDR
1	PRAME	9.300	0.00E+000
2	VGF	8.844	0.00E+000
3	ADAM6	8.344	1.78E-232
4	PAEP	8.237	0.00E+000
5	SPP1	7.723	0.00E+000
6	MIA	6.732	0.00E+000
7	TYR	6.697	0.00E+000
8	S100B	6.455	0.00E+000
9	S100A1	6.172	0.00E+000
10	MLANA	6.017	0.00E+000
11	GPR143	5.894	0.00E+000
12	TSPAN10	5.748	0.00E+000
13	CXCL9	5.743	0.00E+000
14	SNX10	5.713	0.00E+000
15	CCL5	5.577	0.00E+000
16	MMP9	5.454	0.00E+000
17	ABCB5	5.405	0.00E+000
18	PI15	5.366	0.00E+000
19	ELOVL2	5.347	0.00E+000
20	EDN3	5.332	1.56E-171
21	SLC24A5	5.191	0.00E+000
22	LZTS1	5.182	0.00E+000
23	CA14	5.055	0.00E+000
24	SHISA2	5.019	2.37E-319
25	BCAN	5.000	0.00E+000
26	FCGR3A	4.998	0.00E+000
27	03-Sep	4.945	0.00E+000
28	FAM167B	4.941	0.00E+000
29	IGSF11	4.931	0.00E+000
30	TMEM229B	4.889	0.00E+000
31	COL11A2	4.887	0.00E+000
32	SOX10	4.872	0.00E+000
33	LUZP6	4.864	0.00E+000
34	PLP1	4.821	0.00E+000
35	SHC4	4.751	0.00E+000
36	HHATL	4.678	8.74E-181

No.	Gene	logFC	FDR
51	NES	4.379	0.00E+000
52	MEGF10	4.371	0.00E+000
53	TRIM63	4.370	0.00E+000
54	L1CAM	4.349	0.00E+000
55	C1QA	4.340	0.00E+000
56	C1QB	4.293	0.00E+000
57	CD3E	4.288	8.83E-255
58	EFR3B	4.224	0.00E+000
59	TYRP1	4.219	3.59E-170
60	HMCN1	4.217	0.00E+000
61	C1QC	4.212	0.00E+000
62	FREM2	4.207	1.72E-218
63	RNF157	4.166	0.00E+000
64	CSPG4	4.122	0.00E+000
65	BAMBI	4.096	0.00E+000
66	LGALS3BP	4.044	0.00E+000
67	MARCKSL1	4.029	0.00E+000
68	PRH1	4.005	8.84E-288
69	IRF4	4.001	0.00E+000
70	CEACAM1	3.999	0.00E+000
71	ALDH1A3	3.996	0.00E+000
72	GBP4	3.989	0.00E+000
73	AP1S2	3.975	0.00E+000
74	RAB17	3.973	0.00E+000
75	PDE3B	3.968	0.00E+000
76	CYBB	3.966	1.47E-304
77	MPZ	3.948	0.00E+000
78	ITGAL	3.944	0.00E+000
79	IFI6	3.917	0.00E+000
80	SDC3	3.907	0.00E+000
81	FCGR2A	3.905	0.00E+000
82	QPRT	3.896	0.00E+000
83	EXTL1	3.894	0.00E+000
84	BST2	3.891	0.00E+000
85	CDH19	3.883	1.51E-263
86	SH3TC2	3.877	0.00E+000

37	EDNRB	4.640	0.00E+000	87	AKAP6	3.865	0.00E+000
38	GBP5	4.621	0.00E+000	88	B3GNT7	3.822	0.00E+000
39	CRTAC1	4.609	4.15E-296	89	LINGO1	3.803	0.00E+000
40	PAX3	4.605	0.00E+000	90	GMPR	3.769	0.00E+000
41	APOC1	4.578	0.00E+000	91	DBNDD1	3.765	0.00E+000
42	SLAMF7	4.577	0.00E+000	92	PPM1H	3.746	0.00E+000
43	GYG2	4.557	0.00E+000	93	ETV5	3.744	0.00E+000
44	ADCY1	4.535	0.00E+000	94	SNCA	3.735	0.00E+000
45	CD8A	4.513	1.66E-297	95	CNIH3	3.728	0.00E+000
46	HAPLN1	4.495	3.83E-148	96	SERPINE2	3.719	1.30E-254
47	HES6	4.455	0.00E+000	97	GNG7	3.713	0.00E+000
48	PLEKHB1	4.420	0.00E+000	98	PKNOX2	3.708	0.00E+000
49	LEF1	4.417	0.00E+000	99	RGS20	3.705	0.00E+000
50	LYZ	4.401	0.00E+000	100	C2	3.701	0.00E+000

Abbreviations: logFC, log2-fold change; FDR, false discovery rate.

(b-1) Heatmap of gene expressions (z-score) that were significantly upregulated in the melanoma tissues compared to normal skin tissues and affected survival according to the expression level (n=209)

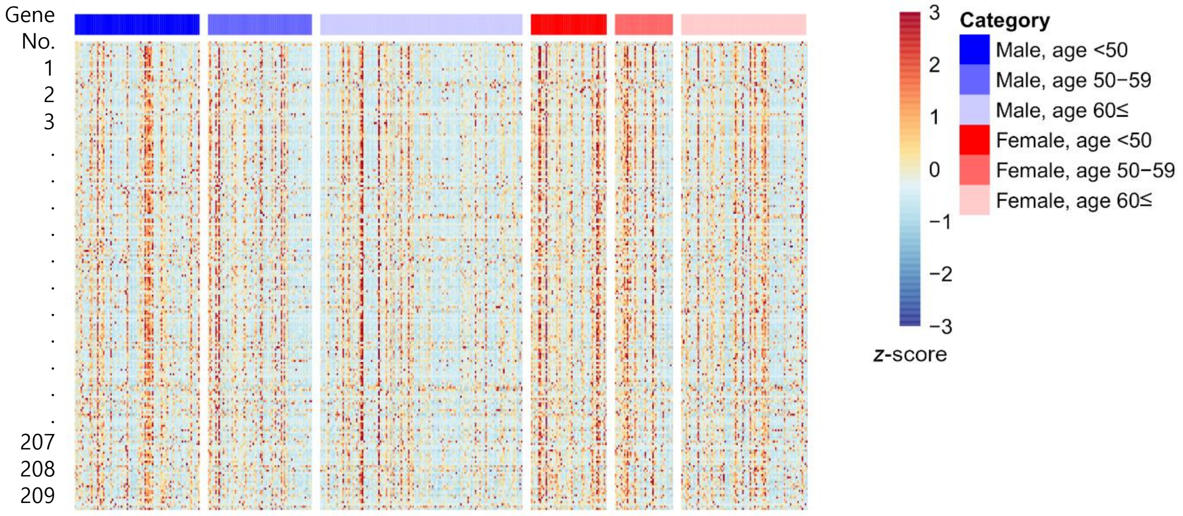

(b-2) Top 100 genes of absolute logFC among the 209 genes

No.	Gene	logFC	FDR	No.	Gene	logFC	FDR
1	ADAM6	8.344	1.78E-232	51	CD6	2.999	4.67E-156
2	GPR143	5.894	0.00E+000	52	CD74	2.990	5.52E-180
3	CXCL9	5.743	0.00E+000	53	IFI27	2.981	4.49E-262
4	CCL5	5.577	0.00E+000	54	HLA-DRA	2.961	7.10E-177
5	FCGR3A	4.998	0.00E+000	55	LAIR1	2.916	1.06E-198
6	GBP5	4.621	0.00E+000	56	CORO1A	2.906	2.81E-250
7	SLAMF7	4.577	0.00E+000	57	AIF1	2.897	2.10E-170
8	CD8A	4.513	1.66E-297	58	TBXAS1	2.894	0.00E+000
9	LYZ	4.401	0.00E+000	59	PTPRC	2.864	7.34E-172
10	C1QA	4.340	0.00E+000	60	HAVCR2	2.864	0.00E+000
11	C1QB	4.293	0.00E+000	61	TTYH3	2.859	0.00E+000
12	CD3E	4.288	8.83E-255	62	HLA-DPA1	2.850	1.79E-238
13	TYRP1	4.219	3.59E-170	63	CD4	2.826	5.75E-268
14	C1QC	4.212	0.00E+000	64	OCA2	2.807	1.76E-730
15	GBP4	3.989	0.00E+000	65	SPN	2.807	1.06E-185
16	CYBB	3.966	1.47E-304	66	SLC25A15	2.793	0.00E+000
17	ITGAL	3.944	0.00E+000	67	CD7	2.792	2.24E-277
18	FCGR2A	3.905	0.00E+000	68	CPNE5	2.791	9.91E-226
19	BST2	3.891	0.00E+000	69	APOL1	2.791	0.00E+000
20	GMPR	3.769	0.00E+000	70	LILRB2	2.784	1.24E-158
21	SERPINE2	3.719	1.30E-254	71	C3AR1	2.724	6.39E-209
22	C2	3.701	0.00E+000	72	PTPRCAP	2.710	1.93E-232
23	WARS	3.655	0.00E+000	73	FAM46C	2.700	9.70E-218
24	SASH3	3.582	1.04E-286	74	FERMT3	2.658	0.00E+000
25	PTPN7	3.532	3.38E-283	75	TAGAP	2.658	3.87E-191
26	OAS2	3.498	0.00E+000	76	CD27	2.652	5.01E-320
27	FPR3	3.484	4.17E-205	77	PIM2	2.646	0.00E+000
28	CTSS	3.444	0.00E+000	78	SEMA6A	2.635	1.15E-227
29	IL2RG	3.436	1.18E-242	79	PSEN2	2.596	0.00E+000

30	CD52	3.375	1.27E-216
31	FCER1G	3.375	3.44E-302
32	HLA-DOA	3.366	8.08E-218
33	TTYH2	3.277	0.00E+000
34	PIK3AP1	3.253	1.65E-267
35	C4A	3.235	0.00E+000
36	CD53	3.228	2.66E-246
37	KLHL6	3.211	5.98E-223
38	LAPTM5	3.200	4.39E-298
39	TYROBP	3.178	9.99E-230
40	PLEK	3.168	2.33E-244
41	IFI30	3.164	0.00E+000
42	MSR1	3.134	1.97E-305
43	SERPINA1	3.133	7.74E-284
44	CST7	3.130	9.11E-188
45	CD14	3.115	0.00E+000
46	SPOCK2	3.112	1.12E-298
47	MPEG1	3.066	4.05E-189
48	ITGB2	3.058	0.00E+000
49	HCST	3.043	0.00E+000
50	RARRES3	3.042	0.00E+000

80	VAV1	2.595	5.20E-146
81	SELPLG	2.589	0.00E+000
82	F5	2.529	1.47E-780
83	LCP1	2.525	2.25E-193
84	IL32	2.521	5.88E-278
85	APOBEC3G	2.506	0.00E+000
86	HLA-DQA1	2.506	1.96E-112
87	WAS	2.483	2.96E-212
88	ARHGAP15	2.463	2.09E-127
89	CDC25A	2.462	3.58E-301
90	ST8SIA4	2.461	2.98E-269
91	HLA-DPB1	2.460	7.21E-215
92	IL27RA	2.450	0.00E+000
93	BIN2	2.449	5.73E-138
94	CD200	2.439	7.28E-185
95	IRF8	2.439	6.78E-118
96	ICAM1	2.422	0.00E+000
97	ABCC2	2.402	2.12E-194
98	LCP2	2.388	1.21E-122
99	CSGALNACT1	2.373	2.53E-254
100	SLC15A3	2.342	0.00E+000

Abbreviations: logFC, log2-fold change; FDR, false discovery rate.

(c-1) Heatmap of gene expressions (z-score) that were significantly downregulated in the melanoma tissues compared to normal skin tissues (n=2381)

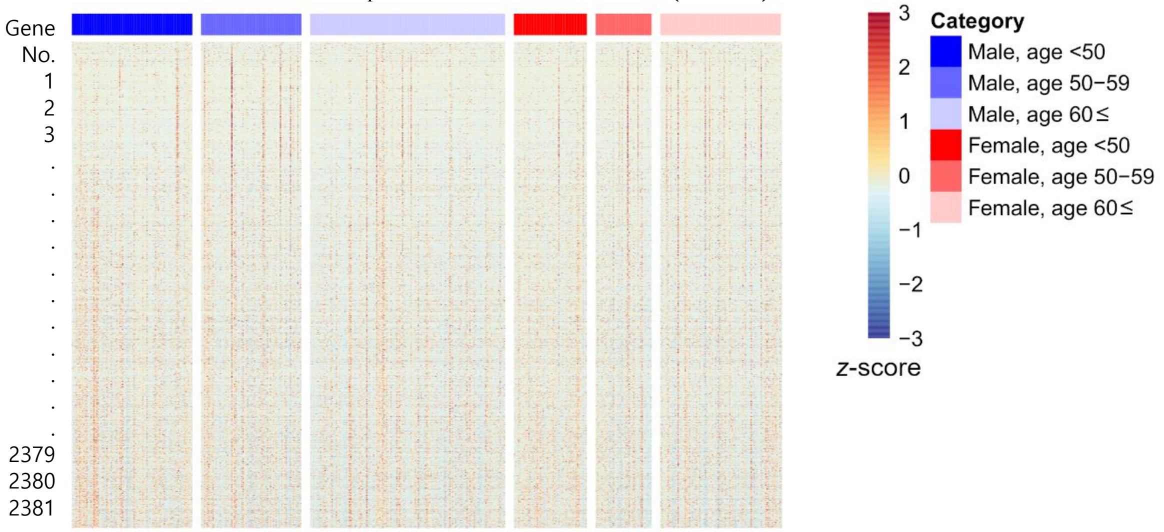

(c-2) Top 100 genes of absolute logFC among the 2381 genes

No.	Gene	logFC	FDR
1	SNORD36A	-15.514	0.00E+000
2	TSSK2	-15.476	0.00E+000
3	UGT1A5	-14.878	0.00E+000
4	CELA3A	-13.085	5.78E-241
5	CLDN22	-12.821	0.00E+000
6	BCYRN1	-12.131	0.00E+000
7	UGT1A3	-12.005	0.00E+000
8	SLC5A2	-11.901	0.00E+000
9	GPR78	-11.621	0.00E+000
10	PSORS1C1	-11.284	0.00E+000
11	SNORA4	-11.033	0.00E+000
12	TREH	-10.999	0.00E+000
13	NKX6-2	-10.994	0.00E+000
14	SNORA70	-10.864	0.00E+000
15	SPATA21	-10.816	0.00E+000
16	OVCH2	-10.646	0.00E+000
17	UGT1A4	-10.491	0.00E+000
18	DNAJC25-GNG10	-10.371	0.00E+000
19	ZACN	-10.279	0.00E+000
20	UGT1A8	-10.194	0.00E+000
21	CELA2A	-10.147	1.80E-285

No.	Gene	logFC	FDR
51	SERPINB10	-8.220	0.00E+000
52	SNORA67	-8.198	0.00E+000
53	GLTPD2	-8.141	0.00E+000
54	KRT2	-8.137	3.26E-219
55	SNORD22	-8.110	0.00E+000
56	CPA1	-8.021	1.95E-215
57	PGA4	-7.995	5.32E-130
58	KRT77	-7.970	4.81E-153
59	DCST1	-7.954	0.00E+000
60	PCDHGB3	-7.944	0.00E+000
61	SYCP3	-7.916	0.00E+000
62	EPB42	-7.901	0.00E+000
63	PNLIP	-7.829	4.44E-134
64	DCD	-7.807	2.73E-110
65	GP2	-7.806	1.25E-225
66	SERPINA12	-7.794	1.57E-170
67	CHP2	-7.768	3.24E-184
68	CACNG8	-7.753	0.00E+000
69	KRTAP13-1	-7.744	5.04E-200
70	SCGB2A2	-7.719	1.16E-128
71	GDF7	-7.668	0.00E+000

22	LHFPL5	-10.132	0.00E+000	72	FOXH1	-7.663	0.00E+000
23	CELA3B	-9.842	6.29E-213	73	LRIT2	-7.624	4.57E-150
24	UGT1A1	-9.792	0.00E+000	74	TNNT2	-7.605	1.32E-318
25	MT4	-9.766	3.53E-251	75	KRTAP10-6	-7.595	2.60E-350
26	KRTAP19-5	-9.541	3.57E-130	76	KRT73	-7.594	7.02E-168
27	LIPF	-9.541	2.83E-139	77	LAMB4	-7.588	0.00E+000
28	TPPP2	-9.534	0.00E+000	78	AMN	-7.577	0.00E+000
29	UGT1A10	-9.457	0.00E+000	79	SERPINB11	-7.550	1.02E-264
30	CLPS	-9.155	5.59E-162	80	KRT26	-7.499	1.10E-106
31	VIL1	-9.076	0.00E+000	81	SNORA48	-7.451	0.00E+000
32	FSCN3	-9.021	0.00E+000	82	CYP3A5	-7.384	0.00E+000
33	PGA5	-9.019	3.81E-204	83	C1orf68	-7.369	1.94E-121
34	TMEM189-UBE2V1	-8.971	0.00E+000	84	LCE5A	-7.358	1.36E-124
35	BEST2	-8.939	1.76E-263	85	FAM110C	-7.340	4.72E-257
36	ARHGEF38	-8.850	0.00E+000	86	FSD2	-7.339	0.00E+000
37	UGT1A9	-8.746	4.07E-283	87	SLC18A2	-7.313	0.00E+000
38	SNORA61	-8.704	0.00E+000	88	KRTAP19-3	-7.261	1.46E-180
39	FAM166A	-8.669	0.00E+000	89	ANGPT4	-7.244	0.00E+000
40	PRSS1	-8.602	8.10E-233	90	PGC	-7.216	1.27E-133
41	PCDHGC4	-8.557	0.00E+000	91	ABO	-7.197	0.00E+000
42	KRT38	-8.541	3.66E-104	92	KRTAP24-1	-7.194	8.22E-170
43	USP50	-8.468	0.00E+000	93	CDHR1	-7.161	2.00E-245
44	PCDHGA8	-8.425	0.00E+000	94	LCE1A	-7.154	2.03E-850
45	FGF22	-8.378	0.00E+000	95	KRTAP11-1	-7.133	2.06E-500
46	CLEC2A	-8.353	3.54E-182	96	PKD2L2	-7.118	0.00E+000
47	FAM153B	-8.332	0.00E+000	97	SERINC4	-7.108	0.00E+000
48	TRIM6-TRIM34	-8.283	0.00E+000	98	PTX3	-7.103	6.99E-223
49	SYT8	-8.255	3.36E-249	99	EME2	-7.099	0.00E+000
50	CYP4F12	-8.236	3.48E-256	100	B3GAT2	-7.091	0.00E+000

Abbreviations: logFC, log2-fold change; FDR, false discovery rate.

(d-1) Heatmap of gene expressions (z-score) that were significantly downregulated in the melanoma Tissues compared to normal skin tissues and affected survival according to the expression level (n=123)

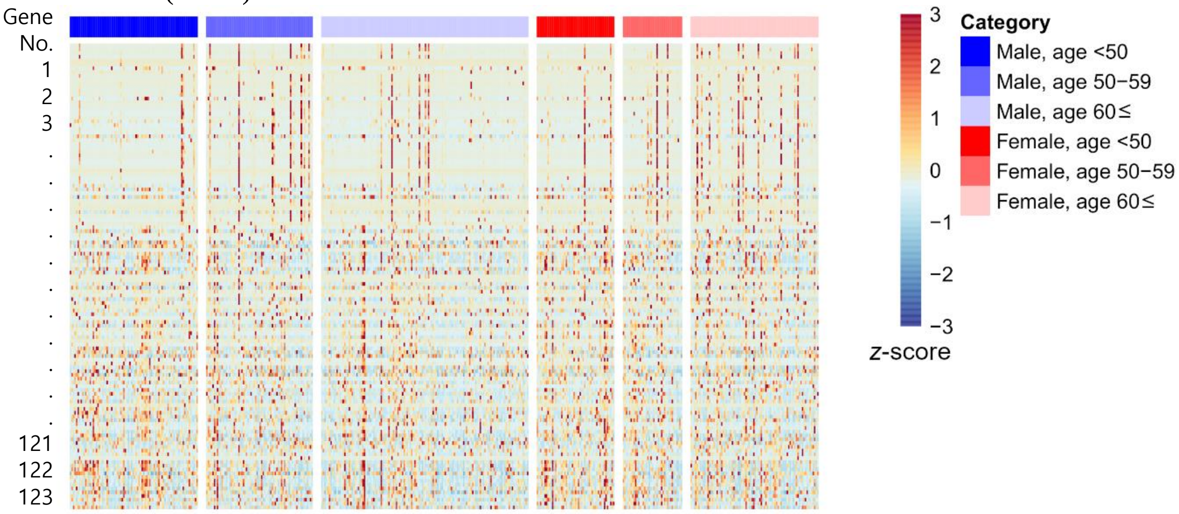

(d-2) Top 100 genes of absolute logFC among the 123 genes

No.	Gene	logFC	FDR	No.	Gene	logFC	FDR
1	C1orf68	-7.369	1.94E-121	51	FERMT1	-3.473	1.91E-140
2	LCE1A	-7.154	2.03E-850	52	FAT2	-3.447	1.79E-122
3	GJB4	-6.664	5.38E-161	53	BCL6	-3.430	0.00E+000
4	POF1B	-6.603	1.47E-168	54	CATSPER2	-3.403	0.00E+000
5	KRT33A	-6.437	6.71E-150	55	KLK5	-3.358	2.96E-530
6	KRT82	-6.434	2.03E-720	56	FGF7	-3.333	1.91E-213
7	HMSD	-6.409	0.00E+000	57	IL18	-3.202	2.79E-158
8	ATP13A5	-6.002	1.90E-116	58	PTGER2	-3.160	3.51E-150
9	LCE2B	-5.847	8.17E-790	59	HSD11B1	-3.042	1.12E-250
10	SLURP1	-5.630	5.54E-960	60	HLA-L	-2.993	0.00E+000
11	PIK3C2G	-5.479	1.41E-960	61	GNB3	-2.955	0.00E+000
12	GGT6	-5.390	1.09E-113	62	CNFN	-2.715	3.20E-630

13	UNC93A	-5.375	3.08E-700	63	PADI3	-2.657	5.88E-380
14	GRHL2	-5.373	1.28E-127	64	BCL11B	-2.634	2.84E-980
15	JSRP1	-5.341	0.00E+000	65	IRX3	-2.622	1.29E-242
16	KPRP	-5.305	1.79E-870	66	MAP3K8	-2.594	1.06E-273
17	LCE2D	-5.154	4.36E-090	67	KRT75	-2.571	6.93E-320
18	PGLYRP3	-5.152	2.61E-680	68	NOD2	-2.538	6.26E-810
19	COL17A1	-5.112	2.23E-130	69	ACSM3	-2.522	2.93E-210
20	ALOX12B	-5.088	1.41E-107	70	RXFP1	-2.519	2.49E-390
21	HSD11B2	-5.051	2.53E-221	71	CUZD1	-2.512	2.73E-308
22	KLRG2	-5.046	3.09E-175	72	WNK2	-2.482	5.92E-680
23	TRIM29	-5.044	9.99E-114	73	SERPINB13	-2.453	4.19E-360
24	RHOV	-4.991	5.42E-169	74	IL18R1	-2.396	3.27E-225
25	TNFRSF25	-4.954	0.00E+00	75	TNFRSF18	-2.389	1.18E-620
26	FAM83E	-4.943	3.50E-199	76	KIAA1324	-2.377	1.28E-900
27	GPR87	-4.884	1.44E-116	77	CP	-2.328	5.52E-109
28	PKP1	-4.871	1.81E-118	78	TNFSF14	-2.322	7.72E-970
29	LCE1E	-4.851	4.06E-640	79	WDR66	-2.265	6.62E-130
30	CRCT1	-4.786	2.21E-890	80	TMEM155	-2.261	5.60E-290
31	ABCA12	-4.780	9.66E-133	81	MAN1A1	-2.241	3.58E-202
32	SERPINB5	-4.771	1.90E-950	82	CPEB3	-2.184	5.92E-178
33	FLG	-4.724	8.48E-145	83	CENPO	-2.175	0.00E+000
34	KRTAP5-11	-4.606	9.52E-250	84	COL4A3	-2.135	4.74E-890
35	CARD18	-4.579	4.58E-590	85	PRDM8	-2.113	7.37E-129
36	CWH43	-4.500	3.98E-670	86	IL7	-2.108	2.84E-208
37	FAM83C	-4.456	2.46E-630	87	SGMS1	-2.092	0.00E+000
38	ARHGEF4	-4.432	5.26E-188	88	RAB40C	-2.057	5.62E-213
39	RGL4	-4.412	0.00E+000	89	BCO2	-2.035	3.09E-186
40	CYP2C18	-4.271	1.45E-880	90	CSF1	-2.031	7.64E-124
41	TRIM69	-4.268	0.00E+000	91	GNAL	-1.980	2.19E-106
42	LY6D	-4.174	3.68E-730	92	ASCL2	-1.979	7.24E-420
43	OTOP3	-4.123	2.43E-660	93	STARD4	-1.943	1.11E-940
44	SULT1E1	-4.023	1.39E-820	94	DAPP1	-1.927	1.30E-490
45	EPS8L1	-3.988	3.69E-202	95	KCNMB1	-1.919	8.04E-740
46	FETUB	-3.950	7.13E-720	96	TNFRSF11B	-1.917	2.40E-380
47	NCCRP1	-3.936	5.71E-103	97	ZEB1	-1.864	1.79E-102
48	NMU	-3.661	1.52E-900	98	FGF2	-1.854	2.31E-650
49	CXCR5	-3.644	5.18E-235	99	TBC1D8	-1.832	2.34E-201
50	C10orf128	-3.488	1.42E-137	100	NFKBIA	-1.815	4.99E-182

Abbreviations: logFC, log2-fold change; FDR, false discovery rate.

Supplementary Figure S2. Comparison of OS in melanoma of the validation set according to signaling expression level. (a) PD-1 signaling, (b) IFN-γ signaling, and (c) IFN-α/β signaling. PD-1, programmed cell death protein 1; IFN, interferon; OS, overall survival.

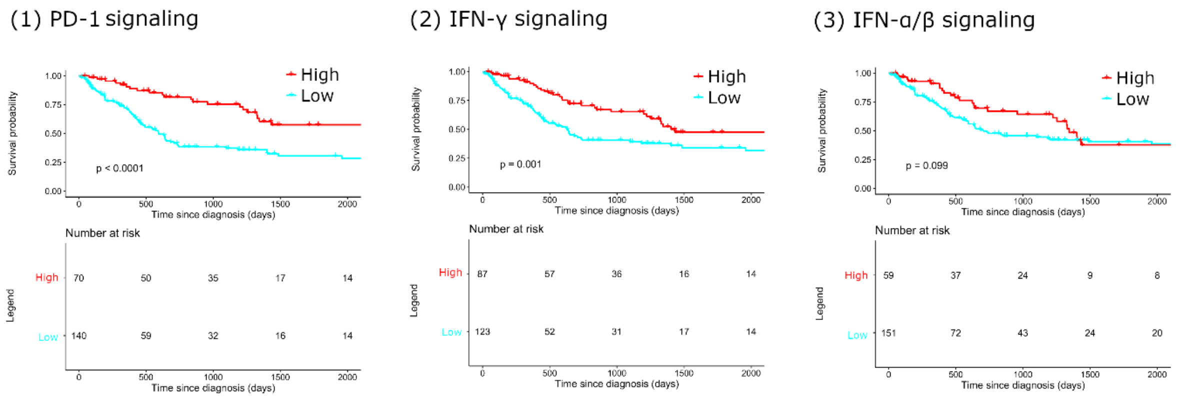

Supplementary Figure S3. Immune signaling according to age and gender in melanoma of the validation set. PD-1, programmed cell death protein 1; IFN, interferon.

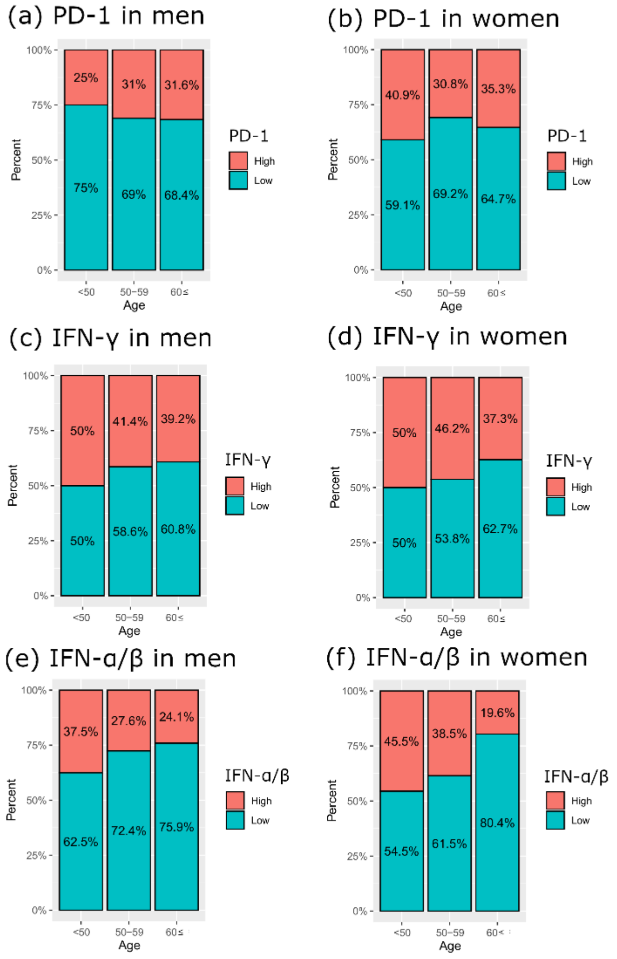

Supplementary Figure S4. Comparison of OS in melanoma of the validation set according to gender and immune signaling. (a) OS in all patients, and (b) OS in patients without high immune signaling. OS, overall survival.

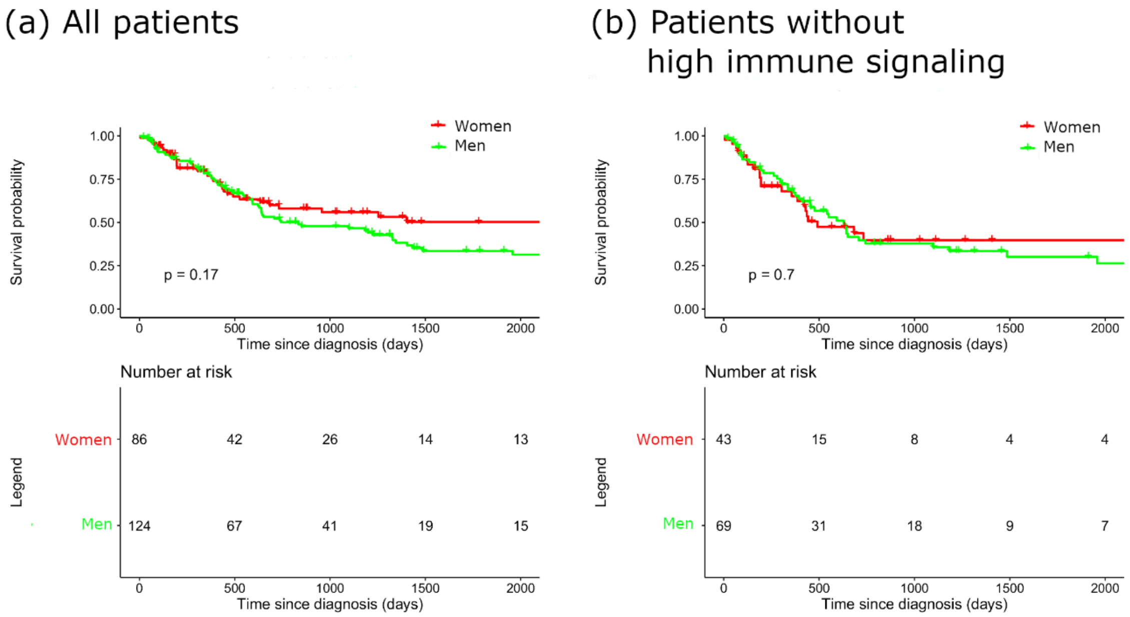

Supplementary Figure S5. Comparison of OS in metastatic melanoma according to signaling expression level. (a) PD-1 signaling, (b) IFN-γ signaling, and (c) IFN-α/β signaling. PD-1, programmed cell death protein 1; IFN, interferon; OS, overall survival.

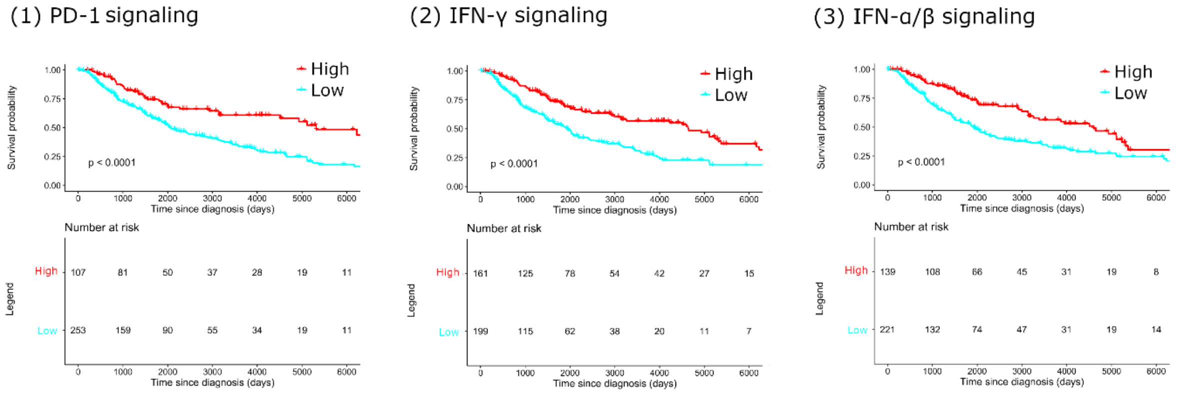

Supplementary Figure S6. Immune signaling according to age and gender in metastatic melanoma. PD-1, programmed cell death protein 1; IFN, interferon.

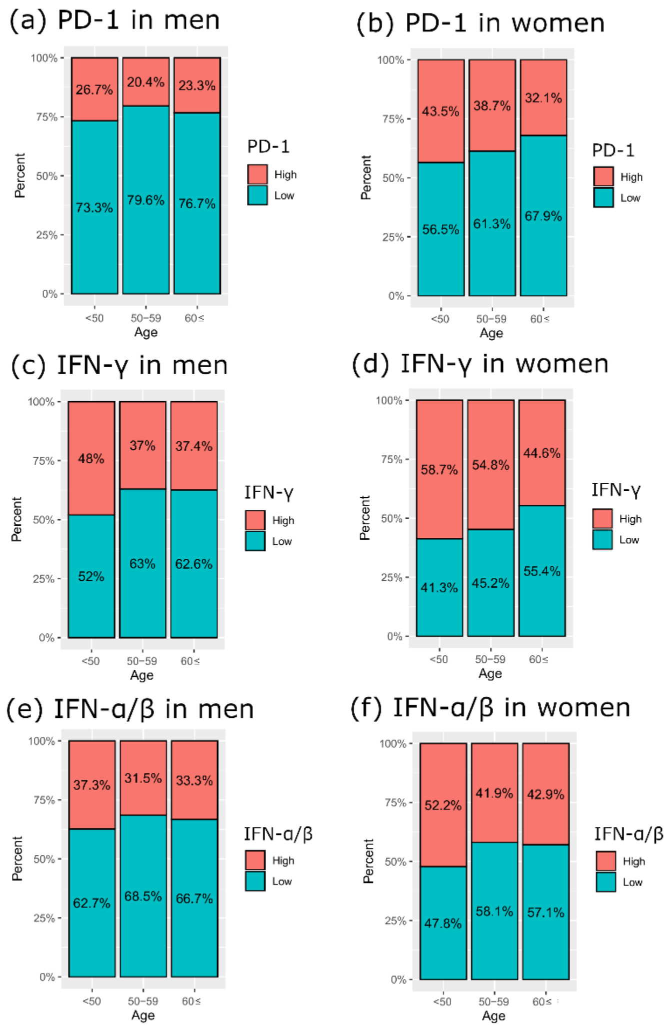

Supplementary Figure S7. Comparison of OS in metastatic melanoma according to age, gender, and immune signaling. (a) OS in patients aged <60 years, (b) OS in patients aged ≥60 years, and (c) OS in patients aged <60 without high immune signaling, and (d) OS in patients aged <60 without high immune signaling. OS, overall survival.

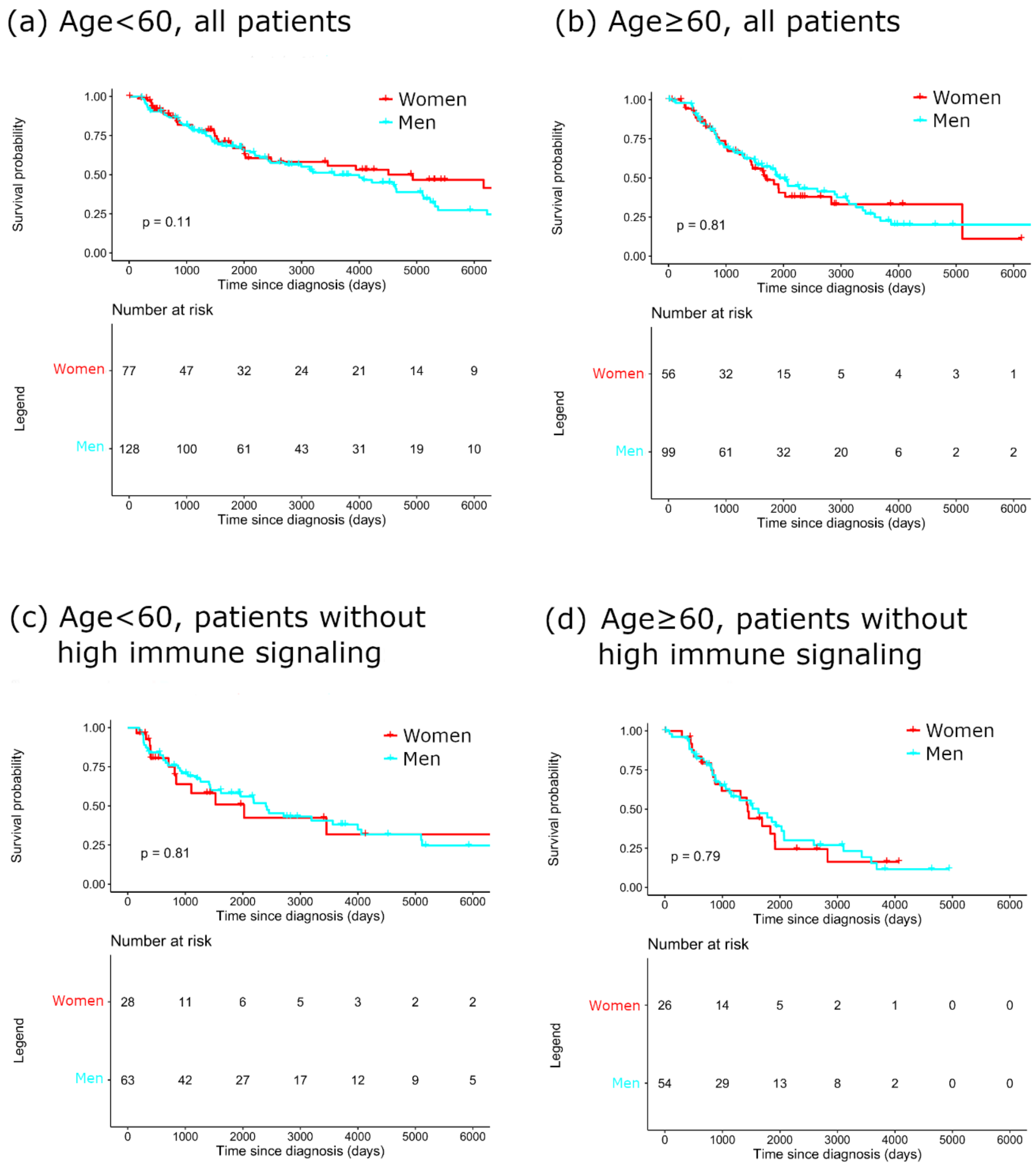

Supplementary Figure S8. Comparison of OS in melanoma according to signaling expression level (differential expression analysis was performed using melanocyte samples). (a) PD-1 signaling, (b) IFN-γ signaling, and (c) IFN-α/β signaling. PD-1, programmed cell death protein 1; IFN, interferon; OS, overall survival.

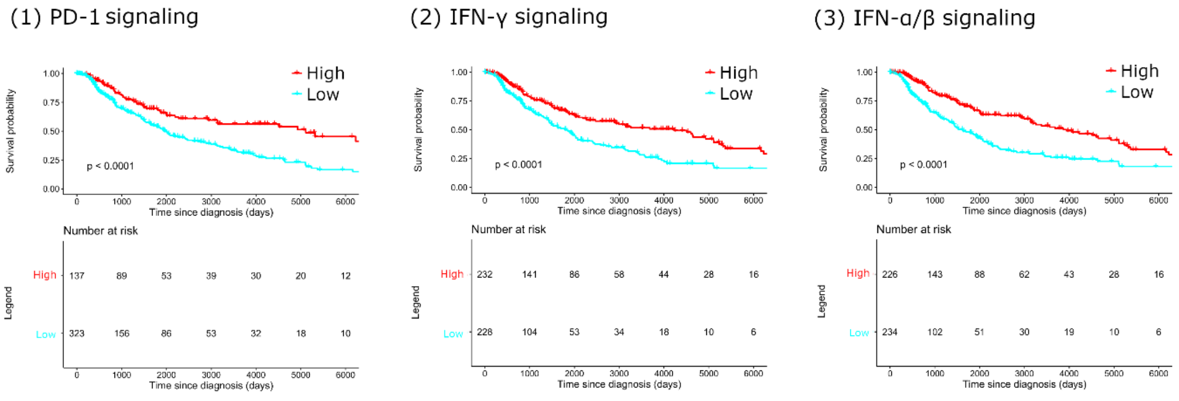

Supplementary Figure S9. Immune signaling according to age and gender in melanoma (differential expression analysis was performed using melanocyte samples). PD-1, programmed cell death protein 1; IFN, interferon.

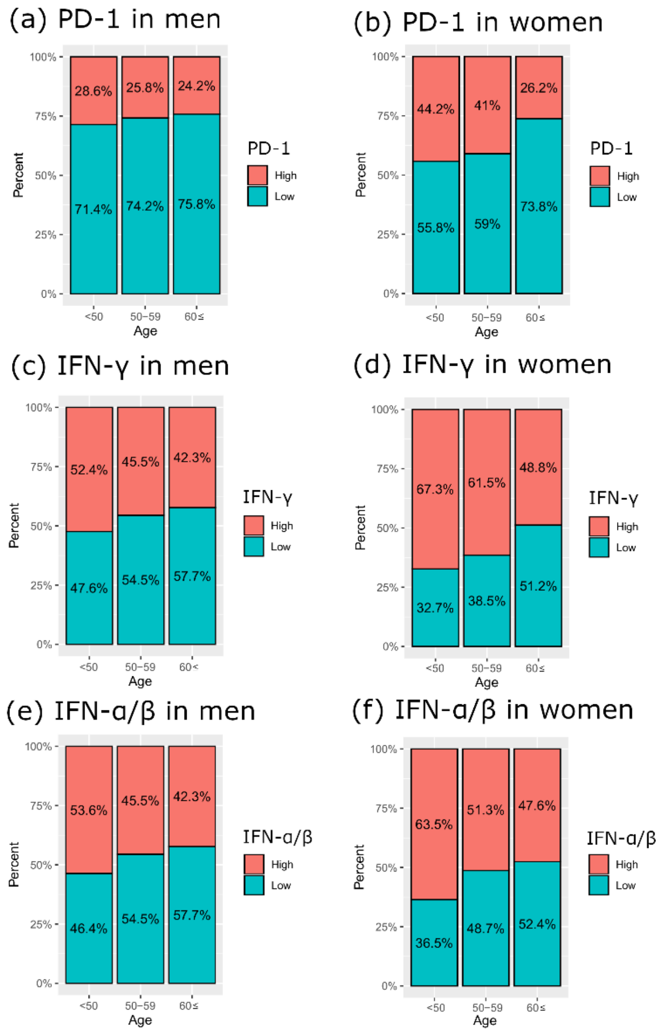

Supplementary Figure S10. Comparison of OS in melanoma according to age, gender, and immune signaling (differential expression analysis was performed using melanocyte samples). (a) OS in patients aged <60 years, (b) OS in patients aged ≥60 years, and (c) OS in patients aged <60 without high immune signaling, and (d) OS in patients aged <60 without high immune signaling. OS, overall survival.

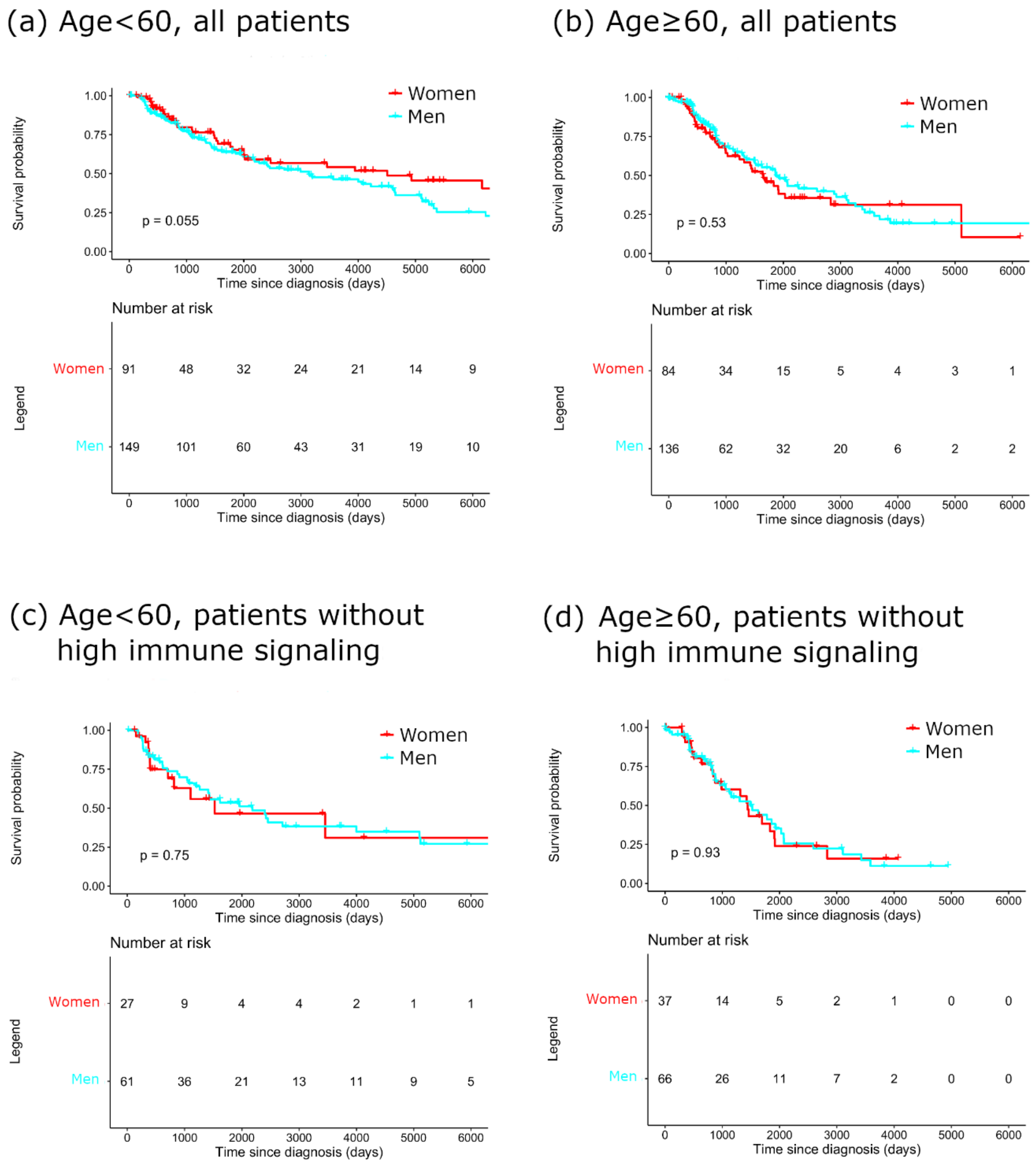

Supplementary Table S1. The 5-year OS according to treatment for the all melanoma patients in the TCGA database

Characteristics	No.	(%)	5-year OS	(95% CI)	P value*
Treatment prior to biopsy					
No	445	(94.7)	57.0	(51.8-62.8)	0.179
Yes	25	(5.3)	75.6	(60.3-94.7)	
Treatment after biopsy†					
No/unknown	219	(46.6)	52.9	(45.0-62.2)	0.390
Yes	251	(53.4)	61.8	(55.5-68.8)	
Treatment before and after biopsy					
No/unknown	450	(95.7)	57.4	(52.2-63.1)	0.344
Yes	20	(4.3)	74.3	(57.1-96.6)	
Treatment before or after biopsy					
No/unknown	214	(45.5)	51.8	(43.8-61.4)	0.235
Yes	256	(54.5)	62.2	(56.0-69.1)	
Chemotherapy					
No	282	(60.0)	58.6	(52.1-66.1)	0.692
Yes	188	(40.0)	57.8	(50.4-66.3)	
Radiotherapy					
No	324	(68.9)	56.3	(49.9-63.5)	0.855
Yes	146	(31.1)	61.7	(53.9-70.6)	

Abbreviations: OS, overall survival; TCGA, The Cancer Genome Atlas; CI, confidence interval.
*Kaplan-Meier survival estimate compared by a log rank test.
†Radiotherapy or chemotherapy.

Supplementary Table S2. The 5-year OS according to treatment for the melanoma patients aged <60 in the TCGA database

Characteristics	No.	(%)	5-year OS	(95% CI)	P value*
Treatment prior to biopsy					
No	224	(92.9)	62.7	(56.0-70.3)	0.667
Yes	17	(7.1)	81.5	(64.5-100.0)	
Treatment after biopsy†					
No/unknown	91	(37.8)	62.2	(51.3-75.4)	0.916
Yes	150	(62.2)	65.4	(57.7-74.1)	
Treatment before and after biopsy					
No/unknown	227	(94.2)	63.4	(56.7-70.8)	0.895
Yes	14	(5.8)	77.1	(57.3-100.0)	
Treatment before or after biopsy					
No/unknown	88	(36.5)	60.3	(49.0-74.1)	0.923
Yes	153	(63.5)	66.2	(58.6-74.7)	
Chemotherapy					
No	123	(51.0)	67.8	(59.0-77.9)	0.191
Yes	118	(49.0)	61.0	(52.1-71.4)	
Radiotherapy					
No	156	(64.7)	63.0	(54.7-72.6)	0.757
Yes	85	(35.3)	66.3	(56.6-77.6)	

Abbreviations: OS, overall survival; TCGA, The Cancer Genome Atlas; CI, confidence interval.
*Kaplan-Meier survival estimate compared by a log rank test.
†Radiotherapy or chemotherapy.

Supplementary Table S3. The 5-year OS according to treatment for the melanoma patients aged ≥60 in the TCGA database

Characteristics	No.	(%)	5-year OS	(95% CI)	P value*
Treatment prior to biopsy					
No	213	(96.4)	50.0	(42.3-59.2)	0.178

Yes	8	(3.6)	62.5	(36.5-100.0)	
Treatment after biopsy†					
No/unknown	126	(57.0)	44.8	(34.4-58.3)	0.365
Yes	95	(43.0)	56.6	(46.5-68.9)	
Treatment before and after biopsy					
No/unknown	215	(97.3)	50.0	(42.3-59.1)	0.318
Yes	6	(2.7)	66.7	(37.9-100.0)	
Treatment before or after biopsy					
No/unknown	124	(56.1)	44.6	(34.1-58.4)	0.242
Yes	97	(43.9)	56.4	(46.4-68.6)	
Chemotherapy					
No	157	(71.0)	50.1	(41.0-61.1)	0.799
Yes	64	(29.0)	52.4	(40.1-68.5)	
Radiotherapy					
No	163	(73.8)	48.3	(39.1-59.6)	0.983
Yes	58	(26.2)	55.5	(43.4-71.1)	

Abbreviations: OS, overall survival; TCGA, The Cancer Genome Atlas; CI, confidence interval.
*Kaplan-Meier survival estimate compared by a log rank test.
†Radiotherapy or chemotherapy.

Supplementary Table S4. Treatment characteristics of all melanoma patients in the TCGA database according to gender.

Characteristics	Male (N=290)	Female (N=180)	P value*
Treatment prior to biopsy			
No	275 (94.8%)	170 (94.4%)	1.000
Yes	15 (5.2%)	10 (5.6%)	
Treatment after biopsy†			
No/unknown	134 (46.2%)	85 (47.2%)	0.905
Yes	156 (53.8%)	95 (52.8%)	
Treatment before and after biopsy			
No/unknown	278 (95.9%)	172 (95.6%)	1.000
Yes	12 (4.1%)	8 (4.4%)	
Treatment before or after biopsy			
No/unknown	131 (45.2%)	83 (46.1%)	0.918
Yes	159 (54.8%)	97 (53.9%)	
Chemotherapy			
No	177 (61.0%)	105 (58.3%)	0.628
Yes	113 (39.0%)	75 (41.7%)	
Radiotherapy			
No	191 (65.9%)	133 (73.9%)	0.084
Yes	99 (34.1%)	47 (26.1%)	

Abbreviation: TCGA, The Cancer Genome Atlas.
*Pearson's chi-squared test.
†Radiotherapy or chemotherapy.

Supplementary Table S5. Treatment characteristics of melanoma patients aged <60 in the TCGA database according to gender.

Characteristics	Male (N=150)	Female (N=91)	P value*
Treatment prior to biopsy			
No	140 (93.3%)	84 (92.3%)	0.967
Yes	10 (6.7%)	7 (7.7%)	
Treatment after biopsy†			
No/unknown	56 (37.3%)	35 (38.5%)	0.970
Yes	94 (62.7%)	56 (61.5%)	

Treatment before and after biopsy			
No/unknown	142 (94.7%)	85 (93.4%)	0.903
Yes	8 (5.3%)	6 (6.6%)	
Treatment before or after biopsy			
No/unknown	54 (36.0%)	34 (37.4%)	0.940
Yes	96 (64.0%)	57 (62.6%)	
Chemotherapy			
No	78 (52.0%)	45 (49.5%)	0.802
Yes	72 (48.0%)	46 (50.5%)	
Radiotherapy			
No	91 (60.7%)	65 (71.4%)	0.120
Yes	59 (39.3%)	26 (28.6%)	

Abbreviation: TCGA, The Cancer Genome Atlas.
*Pearson's chi-squared test.
†Radiotherapy or chemotherapy.

Supplementary Table S6. Treatment characteristics of melanoma patients aged ≥60 in the TCGA database according to gender.

Characteristics	Male (N=137)	Female (N=84)	P value*
Treatment prior to biopsy			
No	132 (96.4%)	81 (96.4%)	1.000
Yes	5 (3.6%)	3 (3.6%)	
Treatment after biopsy†			
No/unknown	77 (56.2%)	49 (58.3%)	0.865
Yes	60 (43.8%)	35 (41.7%)	
Treatment before and after biopsy			
No/unknown	133 (97.1%)	82 (97.6%)	1.000
Yes	4 (2.9%)	2 (2.4%)	
Treatment before or after biopsy			
No/unknown	76 (55.5%)	48 (57.1%)	0.918
Yes	61 (44.5%)	36 (42.9%)	
Chemotherapy			
No	98 (71.5%)	59 (70.2%)	0.958
Yes	39 (28.5%)	25 (29.8%)	
Radiotherapy			
No	98 (71.5%)	65 (77.4%)	0.423
Yes	39 (28.5%)	19 22.6%)	

Abbreviation: TCGA, The Cancer Genome Atlas.
*Pearson's chi-squared test.
†Radiotherapy or chemotherapy.
